# State-wide population screening for Lynch syndrome? Improved ascertainment of at risk families

**DOI:** 10.1186/1897-4287-10-S2-A77

**Published:** 2012-04-12

**Authors:** J Goldblatt, B Iacopetta, B Amanuel, L Schofield

**Affiliations:** 1 Genetic Services and Familial Cancer Program of WA, Australia; 2 School of Surgery, UWA, Australia; 3 Molecular Pathology, PathWest, WA, Australia

## 

We have previously established in a large retrospective study that MSI testing was an effective first screen for the identification of individuals with Lynch syndrome (LS) in colorectal cancer (CRC) patients aged < 60 years. From these findings, MSI and/or IHC screening was recommended for all newly diagnosed CRC patients aged < 60 years in Western Australia, regardless of family history of cancer. We have subsequently evaluated the utility of routine MSI/IHC screening in diagnostic pathology laboratories for the detection of previously undiagnosed individuals and families with LS. From January 2009 to December 2010, 270 tumours were tested for MSI and expression of MLH1, PMS2, MSH2 and MSH6 using IHC. Cases showing MSI and/or loss of expression were also tested for *BRAF* V600E mutation. Seventy cases were found to have MSI, of which 25 were excluded from further investigation as possible LS cases due to the presence of the *BRAF* V600E mutation. The remaining 45 “red flag” cases were eligible for germline testing based on their MSI, IHC and *BRAF* status. From 26 cases tested to date, 11 germline mutations have been found. Nine were from individuals not previously recognized as LS and two were untested members from known LS families. Extrapolation of the mutation incidence (11/26, 42%) to all red flag cases (n=45) suggests approximately 19 mutation carriers in this cohort. This value approximates the number of LS cases that could be expected to arise in the Western Australian population over a two-year period (n=24), assuming that 1% of all CRCs are due to LS. Our preliminary findings following the implementation of state-wide routine MSI and IHC testing in Western Australia indicate that the majority of LS cases are being identified.

